# The impact of sharing physical activity experience on social network sites on residents’ social connectedness:a cross-sectional survey during COVID-19 social quarantine

**DOI:** 10.1186/s12992-021-00661-z

**Published:** 2021-01-11

**Authors:** Yifan Zuo, Yudan Ma, Mu Zhang, Xiaoyuan Wu, Zhanbing Ren

**Affiliations:** 1 School of Management, Jinan University, Guangzhou, 510632 China; 2 Shenzhen Tourism College, Jinan University, Shenzhen, 518053 China; 3 Shanwei Polytechnic, Shanwei, 516600 China; 4 Department of Physical education, Shenzhen University, Shenzhen, 518061 China

**Keywords:** COVID-19, Sharing physical activity experiences on SNS, Social connectedness, Positive self-presentation, Positive feedback

## Abstract

**Background:**

During isolation, sharing physical activity experiences on social network sites (SNS) can enhance individual social connectedness. The objective of the present study was to examine the associations between sharing physical activity experiences on SNS, positive self-presentation, positive feedback, and social connectedness during isolation.

**Methods:**

Based on the Physical Activity Experience Sharing Scale, Social Connectedness Scale, Positive Self-Presentation Scale, and Online Positive Feedback Scale, we collected 460 questionnaires online from across 31 provinces, municipalities and autonomous regions in China. We used multiple linear regression models to investigate the relationship between variables, and used bootstrapping to test for mediation..

**Results:**

During isolation, sharing physical activity experiences was positively associated with social connectedness (b = 0.308, *p* < 0.001), as well as with positive self-presentation(b = 0.956, *p* < 0.001)and positive feedback(b = 0.421, *p* < 0.001). In addition,we found that positive self-presentation showed a significant positive impact on positive feedback (b = 0.563, *p* < 0.001), and that positive self-presentation(b = 0.331, *p* < 0.001) and positive feedback(b = 0.311, *p* < 0.001) were positively associated with social connectedness. Finally, we found an effect on sharing physical activity experiences indirectly through positive self-presentation (b = 0.316, 95% CI: [0.180, 0.463]),and positive feedback (b = 0.131, 95% CI: [0.063, 0.207]) and that the mediation chain between the two also had a significant impact on social connectedness (b = 0.167, 95% CI: [0.088, 0.251]).

**Conclusions:**

During the COVID-19 pandemic, sharing physical activity experiences on SNS can create a positive, healthy, and energetic personal image, gain recognition from others, and establish new interpersonal relationships.

## Introduction

Physical activity not only enhances people’s physical health but also promotes socialization and social connectedness [1]. Social connectedness is a multi-dimensional structure and plays an important role in promoting happiness, self-esteem, and confidence [2]. However, with the outbreak of the new coronavirus pneumonia in 2019(COVID-19), countries around the world quickly enacted restrictions on activities, socializing, and gatherings [3]. Such restrictions have also significantly reduced physical activity: most organized and commercial fitness activities have been suspended, and residents consequently affected the relationship between individuals and people around them, between individuals and society; and continuous isolation may lead to persistent problems in the relationship between people and society [4].

In the context of COVID-19 physical activity is playing a limited role in promoting interpersonal relationships and enhancing social connectedness. However, with the continuous extension of social evacuation and blockade orders, contact with the outside world has turned to social network sites (SNS), that enable communication with others via online entertainment, leisure, and sports activities [5]. Indeed, during the pandemic, SNS usage has increased by 61% [6], from February 2020 to March 2020, global usage of Facebook and Instagram increased by more than 40%. During this same period, messaging rates on Facebook, Messenger, WhatsApp, and Instagram increased by 70%, and the rate of utilization of local SNS (such as WeChat and Weibo) in China increased by 58% [7]. As a result of this, people have found that in this public health emergency, the use of SNS is the best way to promote the healthy behavior of residents, especially when home exercise information intended to reduce residents’ negative emotions and social distancing caused by social isolation is shared and discussed through SNS [8]. It is currently common to publish and share information about personal home exercise and virtual sports on SNS, making it likely that the ability to share a common physical activity experiences in this way enhances social connectedness between individuals [3].

The formation of social connectedness depends on social interaction. Social interaction refers to the many daily communication activities that peopleconsistently engage in, such as pleasing others, saying hello, and learning to communicate. Due to the impact of the COVID-19 offline physical activity exchange has been quickly replaced by sharing physical activity experiences online, enhancing social connectedness among residents [3]. This kind of “offline to online” progression has resulted in people no longer needing to engage in physical contact to achieve the social connectedness of physical activity, but rather being satisfied with doing physical exercise or virtual sports at home, and then sharing and discussing in SNS to enhance their social connectedness with others. The establishment of “sports-lover,” “fitness expert,” “body management master,” and other images can obtain more positive feedback from others [9]. Online positive feedback is the most important form of communication in online social interaction, and is primarily manifested in the timely positive affirmative evaluations exchanged during the process of online interaction [10].

In this study, we focused on residents who have been sharing physical activity experiences on SNS during the period of home isolation of COVID-19 in China. This study emphasized the role of sharing physical activity experiences on SNS and the subsequent consequences for social connectedness among residents during COVID-19-related isolation. We also aimed to deepen our understanding of the role of positive self-presentation and positive feedback of SNS.

## Theoretical background

### Sharing physical activity experiences on SNS during isolation

With the rapid development of SNS (such as Facebook, Instagram, WeChat, and Weibo) people have been given more opportunities for self-expression and social interaction, which has resulted in rich user-created content [11]. Sharing physical activity experiences on SNS mainly refers to the acquisition and exchange of sports knowledge (involving education, culture, competition, and other physical science and technology knowledge), sports topics, and other related information through SNS [12].

During the COVID-19 outbreak, governments around the world strongly recommended that the public stay at home and maintain regular physical activity and daily exercise in a safe home environment [13]. During this period, the home-based exercise was also highly promoted through SNS channels by national organizations. For example, the Australian Olympic Committee encouraged residents to participate in physical activities at home through SNS, and invited professional athletes to provide suggestions and demonstrations for home-based exercise by producing and disseminating a series of home sports videos, to be released on multiple social media platforms [14]. In addition to the benefits of engaging in the exercise itself, residents taking photos and uploading their own home exercise or virtual sports videos may also achieve an inspiring effect [15].

### Personal social connectedness during isolation

Social connectedness is defined as the subjective awareness of being in close relationships with the social world [16]. Based on the core definition of “sense of belonging and interpersonal relationship” from previous studies, the social connection in this study is defined as the intimacy and sense of belonging with friends, family, and the community in the home environment during isolation. In past studies, it is generally believed that SNS can promote people’s social connectedness. People use SNS to keep in touch with family and close friends [17], and it is the case that people who use SNS to maintain existing social relationships have stronger social adaptability and less loneliness [18].

Although the pandemic-related isolation policies are intended to reduce the risk of infection, it may also have negative effects due to social distancing. People currently may lack social connectedness because they cannot go to places where they normally gather [19], especially those who have lower degrees of pro-social behavior. However, research has found a positive correlation between the use of information and communication technology (ICT) and social connectedness, although there are still doubts about the source or content of the exchange of information [20].

### Positive self-presentation in SNS

The concept of self-presentation is based on symbolic interactionism, which refers to the efforts of individuals to show themselves and influence others in order to make others look at themselves based on their wishes [21]. Positive self-presentation refers to the selective presentation of information that can establish a positive image of oneself. This presentation strategy can improve individual self-esteem, positive emotions and life satisfaction [22]. With the increasing popularity of SNS, SNS plays a central role in personal self-expression and social relationship management [23]. As a supplementary form of communication to the existing relationship in real life, the non-immediacy of online communication enables individuals to have maximum control over self-display and expression, thereby making SNS an ideal platform for self-presentation [24]. Positive online self-presentation, as a form of self-expression used by individual SNS, can enable individuals to shape a positive social image on the network according to their own advantages [25].

After the COVID-19 outbreak, people’s preferences for self-presentation on SNS have changed, making it more common to discuss a person’s health and preventive behavior. As a result, sharing this kind of information is beneficial to the public interest and is a socially recognized behavior [5]. For example, athletes spread positive information through SNS, encourage residents to engage in appropriate physical activity at home, check in for daily home sports, and analyze sports information through SNS to enhance social connectedness, thereby establishing a positive image of athletes [5].

### Positive feedback in SNS

Positive feedback is one of the most important forms of social support in SNS and is mainly manifested in the timely positive affirmative evaluation of the other party during the online interaction process [10]. Specifically, it refers to the supportive response received by individuals when they post updates, photos, and other personal information in social applications [26]. Some studies have shown that positive online feedback helps to improve individuals’ social adaptability and social connectedness [27]. However, it is worth noting that some studies have shown that participants with already high positive self-perception will seek out further positive feedback than those with negative self-perception [28]. For example, adolescent girls focus on personal body image, but girls with more idealized body shapes and more participation in physical activity are more eager to receive approval from others or other forms of positive feedback [29]. As such, some people choose a more cautious approach to online self-presentation in order to avoid getting negative feedback on SNS [5].

### Model development, variables, and hypotheses

**Fig. 1:**
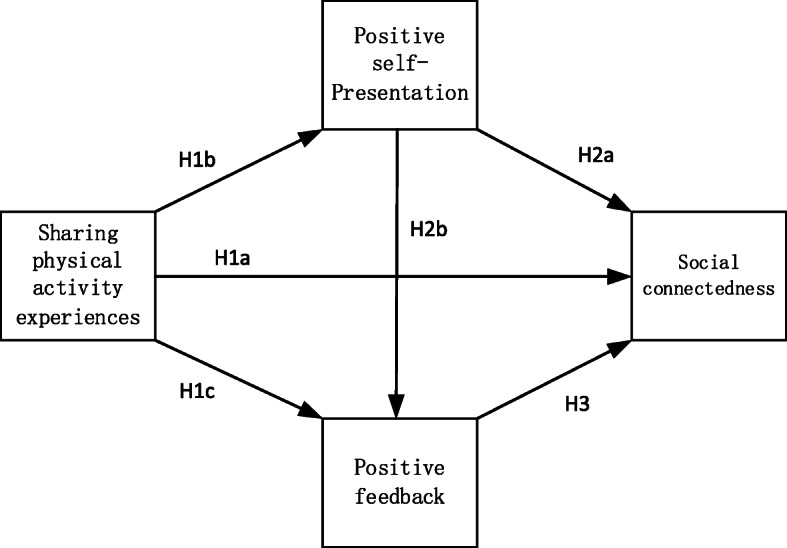
Conceptual mediation model and hypotheses

The conceptual model of mediation effect is shown in Fig. 1, with relevant hypotheses detailed in the following paragraphs.

In the study of online sharing behavior on SNS, enhancing social connectedness is the main motivation for sharing behavior, especially when sharing some physical activity information [30]. In addition, physical activity is inseparable from the information sharing function of SNS [4]. With the outbreak of COVID-19, under the restriction of isolation policy, more and more people choose to exercise at home [31]. At the same time, the frequency of sharing and discussing home exercise and virtual activities on SNS has become increasingly frequent [32]. Combined with previous research results, sharing information and participating in interaction on SNS can enhance social connectedness. During the outbreak, more and more people take sharing physical activity experiences on SNS as a signal to show their health status [33].

Preliminary research indicates that self-presentation may be an important antecedent and consequence of physical activity [34]. Affected by COVID-19, more and more people show their home exercise results or scores of virtual sports on SNS. They regard it as an act of showing their own health and being proactive, which will be recognized by others and is a kind of positive self-presentation [5].. In addition, some studies have shown that sharing information about health behaviors on SNS can receive positive feedback from others, and at the same time, can also create a positive image in others’ mind [35]. We therefore make the following predictions.

H1a Sharing physical activity experiences on SNS during home isolation directly and positively influences social connectedness.

H1b Sharing physical activity experiences on SNS during home isolation directly and positively influences online positive self-presentation.

H1c Sharing physical activity experiences on SNS during home isolation directly and positively influences positive online feedback from others.

SNS has greatly changed the way people communicate, connect and express themselves. Some studies have found that individuals in SNS can create a satisfactory and ideal self-image with the help of positive self-presentation, thereby getting rid of the social distress in real social interactions and enhancing social connectedness [36]. The studies show that when individuals present information related to themselves through self-presentation, they encourage others to give feedback. In the process of presentation and feedback, the two formed a good relationship, and deepened the good interpersonal relationship in the process of repeated circulation [10]. In addition, studies have shown that SNS promote interpersonal communication and maintain peer relationships among adolescents through positive feedback, which is an important factor in improving the quality of friendship [37]. Online positive feedback on SNS can have a positive impact on personal offline intimacy [38]. We therefore make further predictions to investigate these issues.

H2a During home isolation, online positive self-presentation directly and positively influences social connectedness.

H2b During home isolation, online positive self-presentation directly and positively influences positive online feedback from others.

H3 During home isolation, online positive feedback directly and positively influences social connectedness.

In the social pension theory, it is pointed out that the establishment or development of “disclosure-feedback-relationship” is the basic process to promote the establishment and development of interpersonal relationship [39]. Individuals can express themselves through information transmission, and getting positive feedback from others will deepen interpersonal relationships between people. Specifically, individuals can post, share, and discuss positive information related to themselves in SNS, thereby showing a positive image and friends giving positive feedback on the posted information. When individuals receive positive feedback from their friends, they will enhance their social connectedness between themselves and their friends [40]. During pandemic, everyone is keen to discuss a person’s health status and preventive behavior. It is generally recognized that sharing such information is beneficial to the public interest, which is a behavior that can be recognized by others [5]. According to the social penetration theory, SNS will classify people who have the same pursuit of health into one category; in this way, sharing physical activity experiences on SNS will form a new social interaction, and such group of people will think that sharing physical activity experiences can promote self-presentation, where this positive form of self-presentation can be recognized by others and promote the establishment of interpersonal relationship. Such findings lead us to our final set of predictions.

H4 During home isolation, positive self-presentation plays a mediating role between sharing physical activity experiences on SNS and social connectedness.

H5 During home isolation, positive feedback plays a mediating role between sharing physical activity experiences on SNS and social connectedness.

H6 During home isolation, positive self-presentation and positive feedback play a chain-like mediating role in the mechanism of sharing physical activity experiences on SNS and social connectedness.

## Methods

### Procedure and sample

Since the outbreak of COVID-19 in China, all provinces and cities in China successively launched the first-level emergency response plan starting on January 29, 2020, and ending on May 2, 2020, requiring residents to stay at home and reduce travel. During this period, Chinese residents’ social software appeared in the “home school physical education,” “home sports clock in,” “sports-related information about the benefits of physical exercise for the pandemic prevention,” “online sports events,” and other sports related personal status.

Data collection was conducted in July 2020. The survey respondents were recruited using the convenience sampling of non-probability sampling. Questionnaires were distributed using the website https://www.wjx.cn/ through SNS. The first page of the questionnaire informed participants that the survey was anonymous and used for research purposes only. A total of 506 questionnaires were collected from 31 provinces and cities in China. We excluded 46 questionnaires due to quality checks, leaving a total of 460 questionnaires for analysis.

Participants were asked about sharing their physical activity experiences on SNS, positive self-presentation, positive feedback, and social connectedness during the home isolation period. Meanwhile, the information about the participants’ sex, age, household income, education level, employment status, marital status, home ownership, BMI, and other information was recorded. As shown in Table 1, the majority of the participants were male (55.0%), under 30 years of age (55.9%), earned less than 100,000RMB (39.8%), held at least a bachelor’s degree (34.3%), were employed (74.6%), unmarried (61.3%), owned their homes (87.0%), and indicated that they possessed a standard body shape (18.5–23.9 [41]) (75.9%).

**Table 1:** Descriptive statistical results of demographic characteristics

Variable	Mean or percentage (standard deviation)
**Gender**	
Male	55.0%
**Household income**	
Annual income below 100,000	39.8%
Annual income of 100,000-200,000	34.6%
Annual income above 200,000	25.7%
**Marital status**	
Unmarried	61.3%
**Employment status**	
Employed	74.6%
**Age**	
High (below 30 years old)	55.9%
**Education level**	
Junior college and below	33.5%
Bachelor’s degree	34.3%
Postgraduate and above	32.2%
**BMI**	
Standard body shape (18.5–23.9)	75.9%
**Home ownership**	
Yes	87.0%

Note: According to the original data collected before, the dividing line on 30 years old happened to evenly divide samples, where the age data presented normal distribution. Age was set as virtual variable, which was easy to conduct multiple stepwise regression analysis later

### Measures and variables

Based on previous experience, we developed a scale survey process in accordance with standardized procedures. Since the survey was conducted in China, the scale was translated into Chinese in accordance with the reverse translation procedure [42]. The content validity of the items in the scale for measuring each construct was independently evaluated by four scholars and three research assistants for content and comprehensibility of the measurement items. This group also assessed potential item redundancy and quota entries of each construct. We began with a pilot study that took place from July 15 to July 20, 2020, on a group of 50 SNS Chinese platform users between the ages of 18 and 50 years in order to improve performance issues, delete indeterminate or unclear items, refine the survey content and structure, and verify the reliability and validity of the scale.

In order to measure residents’ sharing physical activity experiences on SNS during the period of home isolation, we adapted the Actual Travel Experience sharing [30] to the context of home isolation. It contains four items, each with a frequency of 0 indicating “almost no,” and a frequency of 5 indicating “more than one use/share per exercise.”

In order to measure the sense of social connectedness of residents in the period of home isolation, we administered the Social Connectedness Scale. The scale is a 5-point Likert scaleof eight items, with 1 indicating “strongly disagree” and 5 indicating “strongly agree”. Lower scores indicate a stronger sense of social connectedness. These items describe the general emotional distance between oneself and others [43].

In order to measure the effects of positive self-presentation of sharing physical activity experiences on SNS during the period of home isolation, we adopted the Positive Self-Presentation Scale [22], which contains six items and uses a 5-point Likert scale, with 1 indicating “completely non-compliance,” and 5 indicating “full compliance.” The self-presentation strategy scale developed by the original scale in the study of Facebook measures the degree to which individuals present their positive aspects selectively on SNS. In many previous studies of Chinese samples, the confirmatory factor analysis showed good fit and good aggregation validity [44, 45].

In order to measure the effect of positive feedback of sharing physical activity experiences on SNS during the home isolation period, we used the Online Positive Feedback Scale [26]. This scale measures the frequency of positive feedback from individuals after presenting information on SNS. It contains five items and uses a 5-point Likert scale (1 = “never,” 5 = “frequently”). The scale focuses on the frequency rather than the level of positive feedback, because it is difficult for the general public to judge the level of positive feedback; that is, it is difficult to judge whether the comments of friends are mild positive feedback, moderate positive feedback or strong positive feedback. Therefore, based on the actual situation of sharing physical activity experiences on SNS during the period of home isolation, this study will revise the scale and modify its circumstances and context.

### Data analysis

Data analysis was conducted in three parts. First, the quality of the measurement model was evaluated by checking the reliability and validity of each construct [46]. Cronbach’s alpha was used to check the internal consistency of each construct, and the results showed that the reliability was at an acceptable level. We then performed confirmatory factor analysis to check the overall aggregate validity of the scale, and counted the score information of each construct. To test the main effect of the model, we conducted a series of multiple regression analyses. Finally, we used the bootstrap confidence interval to test for potential mediating effects of positive self-presentation and positive feedback, in addition to the bootstrapping method (5000 iterations) with 95% bias-corrected confidence intervals [47].

## Results

### Assessment of the psychometric properties of the measures

Table 2 shows that Cronbach’s alpha values range from 0.79 to 0.92, and all constructs exceed the threshold of 0.75, indicating that the internal consistency between each scale is acceptable [48]. CFA was performed to analyze the goodness of fit of the constructs used in the model: sharing physical activity experiences, social connectedness, positive self-presentation, and positive feedback. The fitting indexes of the final confirmatory factor analysis model were better, χ^2^ = 461.575, *df* = 224, χ^2^/*df* = 2.061, RMSEA = 0.048, GFI = 0.913, NFI = 0.949, IFI = 0.973, TLI = 0.969, CFI = 0.973. The CR values for all the constructs ranged from 0.81 to 0.92, which exceeded the threshold value of 0.70 [49]. The standardized factor load of each item was greater than 0.5 and less than 0.9. Also, the AVE values from all constructs ranged from 0.50 to 0.60, which exceeded the minimum criterion of 0.50 [50]. This shows that there is a better convergent validity.

**Table 2:** Reliability analysis results of each item

	Mean	SD	CITC	α	AVE	CR
**Sharing physical activity experiences**	3.60	0.79		0.79	0.50	0.81
Every time I do physical activity, I share photos.	3.66	1.00	0.84^**^			
Every time I do physical activity, I share videos.	3.29	1.09	0.85^**^			
Every time I do physical activity, I share Wechat moments, Weibo, QQ space, etc.	3.39	1.07	0.84^**^			
Every time I do physical activity, I share my feeling on Keep, HotBody, FitTime Instant Exercise, Fittime Ruijian Times or other websites.	4.05	0.88	0.59^**^			
**Social connectedness**	2.36	0.74		0.92	0.60	0.92
I feel disconnected from the world around me.	2.32	0.93	0.80^**^			
Even around people I know, I don’t feel that i really belong.	2.42	0.93	0.82^**^			
I feel so distant from people.	2.37	0.93	0.81^**^			
I have no sense of togetherness with my peers.	2.37	0.98	0.82^**^			
I don’t feel related to anyone.	2.32	0.90	0.82^**^			
I catch myself losing all sense of connectedness with society.	2.33	0.93	0.81^**^			
Even among my friends, there is no sense of brother/sisterhood.	2.34	0.89	0.81^**^			
I don’t feel I participate with anyone or any group.	2.39	0.87	0.78^**^			
**Positive self-presentation**	3.65	0.74		0.88	0.55	0.88
I post photos that only show the active and healthy side of me.	3.72	0.93	0.81^**^			
I selectively post photos in which I do physical activity.	3.68	0.92	0.78^**^			
I only write messages that portray my health and pleasure regardless of my actual feelings.	3.54	0.97	0.77^**^			
I use smiling emoticons (i.e., smiley: ☺) a lot in the messages related to physical activity I write regardless of my actual feelings.	3.60	0.95	0.81^**^			
I avoid writing about negative things that happen to me when I update my status related to physical activity.	3.68	0.93	0.79^**^			
When I update my status related to physical activity, I only reveal positive feelings.	3.71	0.89	0.79^**^			
**Positive feedback**	3.67	0.77		0.88	0.60	0.88
Every time I share photos about physical activity on SNS, many friends will give good replies, such as: really beautiful, really handsome, really healthy, awesome, etc.	3.70	0.93	0.84^**^			
Every time I post an idea about physical activity on SNS, I get replies from many friends.	3.58	0.94	0.81^**^			
When I post some physical activity on SNS that makes me proud or happy (such as body shape, competition results), many friends will give blessings through reply	3.72	0.93	0.82^**^			
Every time I post some physical activity on SNS, I encounter something interesting or funny, and many friends will reply to express their interest.	3.63	0.94	0.83^**^			
Every time I ask a question about physical activity on SNS, many friends will answer my question through reply, expressing their concern for me.	3.71	0.91	0.83^**^			

Note: ** indicates that the correlation is significant on the 0.01 level. *SD* standard deviation, *CITC* item-total correlation, *α* Cronbach’s alpha value, *AVE* average variance extracted, *CR* composite reliability of each construct, in which the reverse question has been re-scored

### Assessment of the hypothesized relationships

#### Modeling strategy

As mentioned above, considering the need for intermediary testing and understanding the impact of sharing physical activity experiences on SNS on the social connectedness of residents during the isolation period, the independent variables sharing physical activity experiences, the dependent variable social connectedness, the positive self-presentation and positive feedback of the intermediate variable were regarded as the overall continuous variable, thereby discussing regardless of dimensions. Subsequently, a series of multiple linear regression models were established to statistically test the aforementioned hypotheses. The model results are shown in Table 3.

**Table 3:** Multiple linear stepwise regression analysis

Variable	Model 1	Model 2	Model 3	Model 4
Sharing physical activity experiences	0.776^***^	0.441^***^	0.417^***^	0.323^***^
Positive self-presentation		0.428^***^		0.260^***^
Positive feedback			0.421^***^	0.292^***^
Gender				
Female	0.004	0.010	−0.010	−0.002
BMI				
Standard Body Shape	0.249^***^	0.010^**^	0.171^**^	0.135^*^
Overweight	0.001	0.021	0.005	0.016
Age				
High (above 29 years old)	0.008	0.010	0.005	0.007
Household income				
Annual income of 100,000–200,000	− 0.026	− 0.017	− 0.033	− 0.026
Annual income above 200,000	− 0.005	− 0.003	−0.007	− 0.005
Education level				
Undergraduate	−0.060^+^	−0.049	− 0.046	−0.044
Graduate or above	−0.029	−0.017	− 0.016	−0.012
Employment status				
Employed	−0.017	−0.001	0.000	0.005
Marital status				
Married	−0.000	0.0049	−0.008	−0.003
Home ownership				
Yes	0.041	0.0213	0.036	0.025
City	Controlled	Controlled	Controlled	Controlled
Sample size	460	460	460	460
R^2^	0.863	0.876	0.877	0.880

Note: In order to save table space, the standard error is not given; *** means *P* < 0.001, ** means *P* < 0.01, * means *P* < 0.05, + means *P* < 0.1; the reference item of gender in the model is “male”, the reference item of BMI is “underweight”, the reference item of age is “over 30 years old”, the reference item of household income is “below 100,000RMB”, the reference item of education level is “below undergraduate”, the reference item of employment is “not employed”, the reference item of marital status is “unmarried”, and the reference item of home ownership is “No”

#### Main effects

In order to examine the role of sharing physical activity experiences on SNS, online positive self-presentation and positive feedback on social connectedness during the period of home isolation, Model 1 was constructed and used as the benchmark for subsequent modeling. Model 1 only studied the positive relationship between sharing physical activity experiences on SNS and residents’ social connectedness in the period of home isolation(b = 0.776, *p* < 0.001), thus Hypothesis 1a is established. Model 2 and Model 3 preliminarily tested the effect of online positive self-presentation and positive feedback on social connectedness during the period of home isolation. Model 2 adds an online positive self-presentation of antecedent variables on the basis of Model 1. The results show that online positive self-presentation has a significant impact on social connectedness during the home isolation period(b = 0.428, *p* < 0.001), thus Hypothesis 2a is established. Model 3 adds online positive feedback of antecedent variables on the basis of model 1. The results show that online positive feedback has a significant impact on social connectedness during the period of home isolation(b = 0.421, *p* < 0.001), thus Hypothesis 3 is established.

In addition, in order to further test the relationship between variables, a multi-level regression analysis was carried out for each variable. As shown in Table 4, sharing physical activity experiences has a significant positive impact on social connectedness during the home isolation period (b = 0.308, *p* < 0.001), thus Hypothesis 1a is further verified; sharing physical activity experiences has a significant positive impact on online positive self-presentation (b = 0.956, *p* < 0.001), thus Hypothesis 1b is verified; sharing physical activity experiences has a significant positive impact on online positive feedback (b = 0.421, *p* < 0.001), thus Hypothesis 1c is verified. In Table 4, it is also verified that online positive self-presentation has a significant positive impact on online positive feedback (b = 0.563, *p* < 0.001), thus Hypothesis 2b is established. At the same time, Hypothesis 2a (b = 0.331, *p* < 0.001) and Hypothesis 3 (b = 0.311, *p* < 0.001) were verified again. Combining the results in Tables 3 and 4, the mediating role of online positive self-presentation and positive feedback was preliminarily tested.

**Table 4:** Verification of the relationship between variables

DV	IVs	B	S.E.	***t***-value	***P-value***	95% Confidence interval		Hypothesis
						LLCI	ULCI	
SP	PA	0.956	0 .013	69.312	0.000	0.862	0.913	H1b(S)
	R^2^	0.913	F = 4804.163, *P* < 0.001					
SF	PA	0.421	0.036	11.487	0.000	0.338	0.477	H1c(S)
	SP	0.563	.038	15.378	0.000	.512	.662	H2b(S)
	R^2^	0.947	F = 4055.764, *P* < 0.001					
SC	PA	0.308	0.059	4.884	0.000	0.172	0.403	H1a(S)
	SP	0.331	0.069	4.829	0.000	0.197	0.468	H2a(S)
	SF	0.311	0.068	4.380	0.000	0.165	0.434	H3(S)
	R^2^	0.878	F = 1088.452, *P* < 0.001					

Note: *DV* dependent variable, *IVs* independent variable, *B* Standardized coefficients, *PA* sharing physical activity experiences, *S* Support, *SP* positive self-presentation, *SF* positive feedback, *SC* social connectedness, the following are the same

#### Mediation effects

The previous paper preliminarily tested the mediating role of health values between the amount of physical activity and well-being in the period of home isolation. According to the mediating effect analysis procedure, the mediating effect was further divided based on Hayes’ Model 6 and bootstrap method [51]. This method calculates the direct effect coefficient and the indirect effect coefficient of the intermediary through repeated re-sampling of the original sample, and uses the confidence interval to test whether the coefficient of the mediating effect is significant. The test results are shown in Table 5.

**Table 5:** Regression Coefficients of the Mediation Model

Path	B	S.E.	95% Confidence interval		Hypothesis
			Boot Lower	Boot Upper	
**PA→SP→SC**	0.316	0.073	0.180	0.463	H4(S)
**PA→SF→SC**	0.131	0.037	0.063	0.207	H5(S)
**PA→SP→SF→SC**	0.167	0.041	0.088	0.251	H6(S)

The previous article and verification of sharing physical activity experiences are important predictors of social connectedness. The results showed that under the influence of chain mediation, sharing physical activity experiences directly (b = 0.308, 95% CI: [0.172, 0.403]) had a significant impact on social connectedness. At the same time, sharing physical activity experiences indirectly through positive self-presentation (b = 0.316, 95% CI: [0.180, 0.463]) and positive feedback (b = 0.131, 95% CI: [0.063, 0.207]) also had a significant impact on social connectedness. Therefore, during the period of home isolation, positive self-presentation and positive feedback played a partial mediating role in the mechanism of sharing physical activity experiences on SNS on social connectedness.

Since the study model is a chain-type multiple mediation model, involving an interactive relationship between two mediating variables, the multiple mediating variables show sequential characteristics, forming the intermediary chain, so further discussion and verification are needed. First, in Model 4 of Table 3, on the basis of Model 1, online positive self-presentation and positive feedback of the antecedent variables were added at the same time. The results show that positive self-presentation and positive feedback concurrently have a significant impact on social connectedness. Meanwhile, the coefficient of sharing physical activity experiences in Model 4 can be seen to have decreased by 0.453 units compared to Model 1, which was still statistical Significant in the sense. This shows that after adding the online positive self-presentation and positive feedback of the antecedent variable, the positive effect of sharing physical activity experiences on the social connectedness of residents during the isolation period still exists, and the effect is slightly reduced, thus Hypothesis 6 is initially established. After further verification through the bootstrap method, sharing physical activity experiences indirectly through the mediation chain of online positive self-presentation and positive feedback (b = 0.167, 95% CI: [0.088, 0.251]) also has a significant impact on social connectedness.

## Discussion

This study found that during the outbreak of COVID-19, Chinese residents were able to avoid social isolation and alleviate social distancing through sharing physical activity experiences on SNS. The research results show that sharing physical activity experiences on SNS can influence all types of social relationships, thus suggesting that physical activity still has a socializing benefit during isolation period [1], and sharing physical activity experiences can have a great impact on residents’ social connectedness [52]. In the inter-research, it is proposed that users post and share personal status on their own SNS, so as to better present a positive social image in front of others [53]. However, due to the stimulation of the pandemic situation, people’s preference for self-presentation on SNS has been changed, and people prefer to establish their positive social image by transmitting a healthy and energetic state or behavior [54]. Our results support this hypothesis and find that individuals gain positive self-presentation on SNS by showing the results of their home-based exercises or the scores of virtual sports on SNS. We also found evidence that the desire for positive feedback is one of the intrinsic motivations for most people to participate in physical activity [55]. Taking motivation as the starting point, this study further elucidates the connection between sharing physical activity experiences, positive self-presentation, and positive feedback, and expands the relevant theories of SNS research.

As technology develops, people are more willing to extend and augment their interpersonal relationships in reality through self-presentation and positive interpersonal interaction on SNS. SNS can help individuals get rid of the social distress in real social interactions [36]. Particularly during the pandemic, people will try their best to keep the image consistent with the social image in order to obtain praise and pleasant comments from others, thereby alleviating the impact of social isolation [5]. This study validates the previous research from an empirical perspective [38]. We found that positive self-presentation of individuals on SNS can result in obtaining positive feedback. This is consistent with past work that has found that individuals can form good relationships in the process of positive self-presentation and positive feedback, as well as deepen interpersonal relationships in the process [10].

Our model validation shows that residents can establish positive self-image and obtain positive feedback from others through sharing physical activity experiences, thereby enhancing social connectedness. This result suggests that individuals can reveal and present themselves through information transmission and obtain positive feedback from others, deepening interpersonal relationships [40]. During the isolation period, sharing physical activity experiences can satisfy personal online positive self-presentation, making it more likely that people who are keen on pursuing health and vitality will form new social interactions, thereby breaking the social connectedness barrier caused by social isolation [5].

In addition, sharing physical activity experiences often results in praise from others or other forms of positive feedback. In a period of social distancing and isolation, online status related to health, vitality, and pandemic prevention makes it easier to obtain positive feedback, facilitating positive online interaction [40]. In previous studies, it was found that positive self-presentation and positive feedback would form a causal relationship [10]. In contrast to previous studies [39], we found that positive self-presentation is more conducive to shaping a positive self-image. Although actual negative information may be hidden, it is more conducive to the establishment of social relationships, judging from the situation at that time [5]. We additionally found that self-presentation was positively correlated with positive feedback [10], and that given that positive feedback, as the main form of emotional support in the network environment [56], is more likely to be praised [26], positive feedback from online friends can also enhance social connectedness [39].

### Limitations and future research directions

One limitation concerns sample selection. We used a convenience sampling method that limits representativeness of the results, and potentially leads to problems related to statistical sample or selection bias. Future research should examine whether our findings are replicable in larger samples. In addition, more cross-cultural and cross-border samples would benefit our understanding of the generalizability of the results.

Second, our methods, tools, and techniques of data collection were limited. This study used the Internet to distribute questionnaires, resulting in the analysis and discussion of time cross-sectional data. However, then again Internet questionnaires cannot provide face-to-face guidance, leading to possible deviations in data collection. At the same time, the problem of excessive use of mobile phones was ignored in the design of the study; thus social isolation and increased Internet usage may facilitate anxiety and depression [57].

Finally, this study found that BMI have an impact on social connectedness, but the reason cannot be explained due to space limitations. As mentioned in the research of Jong & Drummond (2016), adolescent girls are more likely to pay more attention to individual body image. The fitter body girls have or the more sports activities they take, the more positive feedback they want from others. Various social media are penetrating our daily life, whilst they have built up a concept of “Body Environment”. It refers to the perception of body figure generated from the contents published by users of social media, which mainstreams specific body figure with certain standards on the Internet. In this paper, we hope that in the future “Body Environment” as a variable will be tested how it regulates the relationship between sharing physical activity experiences on SNS and social connectedness.

## Conclusion

This study provides empirical evidence that during the isolation period, individuals sharing physical activity experiences on SNS establish a positive social image and obtain positive feedback from others, thereby enhancing social connectedness. This study highlights the importance of sharing physical activity experiences on SNS and provides suggestions for facilitating the response to catastrophic public health challenges. In addition to home-based exercise or virtual sports, residents should also participate more in discussions on related topics on SNS. During the COVID-19 pandemic, sharing physical activity experiences on SNS can create a positive, healthy, and energetic personal image, allow individuals to gain recognition from others, and establish new interpersonal relationships. Therefore, in addition to advocating home-based exercises, governments at all levels in various countries would do well to advocate for the sharing of home-based exercise or virtual sports on SNS, in order to alleviate the negative consequences of pandemic-related social isolation.

## Data Availability

The raw data supporting the conclusions of this manuscript will be made available by the authors to any qualified researcher.
